# Lignocellulosic Fermentation of Wild Grass Employing Recombinant Hydrolytic Enzymes and Fermentative Microbes with Effective Bioethanol Recovery

**DOI:** 10.1155/2013/386063

**Published:** 2013-09-09

**Authors:** Saprativ P. Das, Arabinda Ghosh, Ashutosh Gupta, Arun Goyal, Debasish Das

**Affiliations:** Department of Biotechnology, Indian Institute of Technology Guwahati, Guwahati, Assam 781039, India

## Abstract

Simultaneous saccharification and fermentation (SSF) studies of steam exploded and alkali pretreated different leafy biomass were accomplished by recombinant *Clostridium thermocellum* hydrolytic enzymes and fermentative microbes for bioethanol production. The recombinant *C. thermocellum* GH5 cellulase and GH43 hemicellulase genes expressed in *Escherichia coli* cells were grown in repetitive batch mode, with the aim of enhancing the cell biomass production and enzyme activity. In batch mode, the cell biomass (*A*_600 nm_) of *E. coli* cells and enzyme activities of GH5 cellulase and GH43 hemicellulase were 1.4 and 1.6 with 2.8 and 2.2 U*·*mg^−1^, which were augmented to 2.8 and 2.9 with 5.6 and 3.8 U*·*mg^−1^ in repetitive batch mode, respectively. Steam exploded wild grass (*Achnatherum hymenoides*) provided the best ethanol titres as compared to other biomasses. Mixed enzyme (GH5 cellulase, GH43 hemicellulase) mixed culture (*Saccharomyces cerevisiae, Candida shehatae*) system gave 2-fold higher ethanol titre than single enzyme (GH5 cellulase) single culture (*Saccharomyces cerevisiae*) system employing 1% (w/v) pretreated substrate. 5% (w/v) substrate gave 11.2 g*·*L^−1^ of ethanol at shake flask level which on scaling up to 2 L bioreactor resulted in 23 g*·*L^−1^ ethanol. 91.6% (v/v) ethanol was recovered by rotary evaporator with 21.2% purification efficiency.

## 1. Introduction

Rapid diminution in the accessibility of fossil fuels poses a serious need for sustainable development of alternative energy source. Depletion of oil supply reserves as well as rise in the greenhouse gas emission has glimmered renewed interest in fuel production from renewable resources. To that end, ethanol fermentation from lignocellulosic substrates has been gaining significant concern in the scientific community. Ethanol as a fuel has several advantages over fossil fuels such as greater air-fuel ratio, higher energy density, and added specific energy with heat of vapourization [1]. As ethanol has a high octane number than petrol, no preignition occurs on use of fuel ethanol. Hence, broad use of ethanol is being done as an economical fuel additive with gasoline [1]. 

The structural conformation of various agricultural residues (leafy biomass) shows that the percent fraction of cellulose is at maximum followed by hemicellulose and lignin [2]. Albeit, being the most abundant renewable resource available, its rigid structure, and its crystalline nature, prevents the efficient utilization of lignocellulose for hydrolysis [2]. Consequently, an effective pretreatment strategy is obligatory for the liberation of the cellulose and hemicellulose from the lignin seal so as to render it accessible for a subsequent hydrolysis step. To date, a fair number of readily available pre-treatment techniques are reported in the literature [3]. Physical pretreatment, often called size reduction, breaks down the substrate physically. Chemical pretreatment disrupts chemical bonds aiding in enhanced enzymatic attack to the plant polymers [3]. Use of microbial enzymes for hydrolysis of lignocellulosic biomass has several merits compared to thermochemical pretreatment such as low energy inputs, modest hardware demands, no generation of environment damaging waste products, and absence of hazardous chemicals. Lignocellulose degrading fungal enzymes have been in use at industrial level for more than three decades. However, the main drawback is the high cost of the commercially available *Trichoderma reesei* cellulolytic enzymes [4]. The cellulosome of *Clostridium thermocellum *is known to have one of the highest rates of cellulose utilization till date reported, displaying a 50-fold higher specific activity than the *T. reesei *system against crystalline cellulose [5]. In a simultaneous saccharification and fermentation (SSF) process for bioethanol production employing Jamun (*Syzygium cumini*) leafy substrate, the recombinant *E. coli *BL21 cells having the recombinant cellulase, the full length gene *Ct*Lic26A-GH5-CBM11 from *Clostridium thermocellum* is informed to have improved cellulolytic activity [6] than the commercial enzymes.

SSF studies from lignocellulosic biomass such as forestry wastes, corn stalk and cobs, rice, and wheat straw expending naturally isolated cellulase have been reported [7, 8], but scant information is obtainable on the use of leafy biomass and also of recombinant enzymes for hydrolysis during saccharification. Northern India has rich plantation of trees like jamun (*Syzygium cumini*), asoka (*Saraca indica*), bamboo (*Bambusa dendrocalamus*), poplar (*Populus nigra*), and eucalyptus (*Eucalyptus marginata*). Easy and ample accessibility of leaves from these trees envisaged interest in exploiting them as substrate for the making and retrieval of many valuable products such as bioethanol [9, 10]. The degree to which the lignocellulosic component of this biomass becomes available to the enzyme depends on the pretreatment category employed. Besides high enzyme activity and optimum temperature, type of cellulolytic enzymes and type of fermentative microbes play an important role in increasing ethanol yield from dried leafy substrates.* Saccharomyces cerevisiae* possesses the intrinsic ability of utilizing various substrates for ethanol production apart from high ethanol tolerance and endurance to metabolic inhibitions. *Candida shehatae* has key enzymes, xylitol dehydrogenase and xylose reductase, enabling it to metabolize pentose sugars for ethanol production through the pentose phosphate pathway [11]. Distillation, rotary vacuum evaporation, and pervaporation are some of the commonly used fermentation product recovery processes [12].

In the present study, an effort was made to improve the activities of recombinant hydrolytic enzymes by using repetitive batch strategy and thereby escalate the ethanol yield by using different combinations of hydrolytic enzymes and fermentative microbes in SSF trials with subsequent ethanol recovery by rotary vacuum evaporation.

## 2. Materials and Methods

### 2.1. Reagents, Chemicals, and Substrates

Carboxy methyl cellulose (CMC), sodium acetate, LB medium, ampicillin, kanamycin, glucose, yeast extract, potassium dichromate, sodium carbonate, sodium bicarbonate, sodium potassium tartrate, sodium sulphate, copper sulphate, ammonium molybdate, sodium arsenate, phosphoric acid, ethanol, and Coomassie brilliant blue G-250 were purchased from Himedia Pvt. Ltd., India. The leafy substrates of various agricultural and forest residues such as jamun (*Syzygium cumini*), neem (*Azadirachta indica*), asoka (*Saraca indica*), bamboo (*Bambusa dendrocalamus*), poplar (*Populus nigra*), eucalyptus (*Eucalyptus marginata*), mango (*Mangifera indica*), and wild grass (*Achnatherum hymenoides*) were provided by Professor Dinesh Goyal, Department of Biotechnology and Environmental Sciences, Thapar University, Patiala, Punjab, India. The leafy substrates were washed thrice with water to remove adhering dust particles, dried, and finally grinded in a mixer grinder to 1 mm mesh size.

### 2.2. Microorganisms and Culturing Conditions

The recombinant *E. coli* BL21 (DE3) cells were transformed using family 5 glycoside hydrolase (GH5) gene from *Clostridium thermocellum* inserted in an expression vector pET21a and expressed earlier [13, 14]. Recombinant cellulase (GH5) is now commercially available at NZY Tech, Lda, Lisbon, Portugal. The recombinant *E. coli* BL21 (plysS) cells [15] harbouring family 43 glycoside hydrolase (GH43) gene from *Clostridium thermocellum* inserted in an expression vector pET28a(+) were expressed earlier [16]. These cells were used as a source of recombinant hemicellulase (GH43) enzyme. Both these *E. coli* BL21 cells were maintained in LB medium as glycerol stock at −80°C in our laboratory. 

The predominantly aerobic fermentative microbes,* Saccharomyces cerevisiae *(NCIM no. 3215) and *Candida shehatae* (NCIM no. 3500), were procured from National Chemical Laboratory (NCL), Pune. *S. cerevisiae* and *C. shehatae* were maintained on MGYP slants (5 mL) containing malt extract (0.3 g·100 mL^−1^), glucose (1 g·100 mL^−1^), yeast extract (0.3 g·100 mL^−1^), and peptone (0.5 g·100 mL^−1^) [17] at 4°C. One loopful of these slant cultures was further inoculated into GYE broth medium containing glucose (1 g·100 mL^−1^) and yeast extract (0.1 g·100 mL^−1^) supplemented with KH_2_PO_4_ (0.1 g·100 mL^−1^), (NH_4_)_2_SO_4_ (0.5 g·100 mL^−1^), and MgSO_4_·7H_2_O (0.05 g·100 mL^−1^), and incubated at 30°C, 120 rpm for 48 h before introducing into fermentation media. Aliquots measuring 1 mL from each of actively growing cultures of *S. cerevisiae *(3.6 × 10^8^ cells·mL^−1^) and *C. shehatae *(2.9 × 10^7^ cells·mL^−1^) were aseptically added to 100 mL fermentation medium. 

### 2.3. Production of Recombinant Cellulase (GH5) and Hemicellulase (GH43)

For production of GH5 cellulase, 50 *μ*L of the *E. coli* BL21 (DE3) culture from glycerol stocks was inoculated into 5 mL of LB medium containing 100 *μ*g·mL^−1^ ampicillin and incubated at 37°C for 16 h at 180 rpm. 1% (v/v) of the culture inoculum was transferred to 250 mL of LB medium in 500 mL flask containing 100 *μ*g·mL^−1^ ampicillin with incubation at 37°C, 180 rpm till the culture reached to midexponential phase (*A*_600 nm_ = 0.6). Isopropyl-*β*-D-thiogalactopyranoside (IPTG) (1 mM final concentration) was added to this midexponential phase culture followed by further 8 h incubation for protein induction [16]. Finally, 1% (v/v) of this culture was inoculated into LB production medium. In case of *E. coli* BL21 (plysS) cells encompassing GH43 hemicellulase, 50 *μ*g·mL^−1^ kanamycin was castoff as a selective marker [16]. The production process was the same as that for GH5 cellulase except after IPTG induction at midexponential phase, the cultivation conditions were 24°C and 200 rpm.

### 2.4. Batch and Repetitive Batch Strategy for Recombinant Hydrolytic Enzymes (GH5 Cellulase and GH43 Hemicellulase) Production

One (%, v/v) of the *E. coli* BL21 (DE3) cells harbouring GH5 cellulase gene was transferred to 250 mL of LB medium in 500 mL flask containing 100 *μ*g·mL^−1^ ampicillin and was incubated at 37°C, 180 rpm. Similarly, 1% (v/v) of the *E. coli* BL21 (plysS) cells harbouring GH43 hemicellulase gene was transferred to 250 mL of LB medium in 500 mL flask containing 50 *μ*g·mL^−1^ kanamycin and was incubated at 37°C, 180 rpm. IPTG (1 mM final concentration) was added to the media till the culture reached to mid-exponential phase (*A*_600 nm_ = 0.6) for the induction of cellulase and hemicellulase genes expression, respectively. *E. coli* BL21 (plysS) cells were maintained under growth conditions of 24°C and 200 rpm after IPTG induction unlike 37°C and 180 rpm for *E. coli* BL21 (DE3) cells. The dynamic profiles of enzyme activities and growth (*A*_600 nm_) for both of the *E. coli* cells were monitored separately by collecting samples at every 1 h interval. Both the cells were cultivated separately in repetitive batch mode for enhancing the biomass productivity and enzyme activity. During repetitive batch mode, the first step remained similar as followed in batch mode, and then approximately, there was removal of 247 mL of medium when the cells touched late log phase or early stationary phase. This was centrifuged, and cell mass was further processed for the isolation of enzymes separately. To remaining broth, 247 mL of fresh medium was added. Similar practice was followed in consecutive three batches where the remaining 3 mL of medium comprising induced cells was castoff as inoculum. Repetitive batch cultivation mode was used for maintaining the ideal conditions supporting the growth of recombinant *E. coli* cells and thus the maximum production of cellulase and hemicellulase. Together for batch and repetitive batch approach, the recombinant intracellular cellulase and hemicellulase enzymes were isolated using sonication. Each of the *E. coli* cells was collected separately by centrifugation (4°C, 7,426*g*, 15 min) and was resuspended in 50 mM sodium phosphate buffer adjusted to a pH 7.0. The recombinant enzymes (GH5 cellulase and GH43 hemicellulase) were expressed as soluble proteins. The cell extract containing each of the soluble enzymes was separately sonicated in an ice bath for 15 min followed by centrifugation (4°C, 15,493*g*, 30 min). Each of the supernatants collected containing separately the crude enzymes (GH5 cellulase and GH43 hemicellulase) was subjected to enzyme activity analysis by determining reducing sugar using methods of Nelson and Somogyi [18, 19].

### 2.5. Pretreatment of Substrates

The finely powdered leafy substrates of jamun, neem, asoka, bamboo, poplar, eucalyptus, mango, and wild grass were subjected to two pretreatment strategies, namely, steam explosion and alkali pretreatment using NaOH.

#### 2.5.1. Steam Explosion

One gram of the dry grinded substrate was taken in 100 mL Erlenmeyer flask. In an autoclave, the flask was kept at 121°C and 15 psi for 1 h. The autoclave was exposed to sudden steam depressurization by completely opening the steam exhaust valve, intending to gain maximum quantity of fermentable sugars in the least treatment time [20].

#### 2.5.2. Alkali (NaOH) Pretreatment

One gram of the powdered substrate was retained in a 100 mL Erlenmeyer flask, adding 20 mL of 0.5 M NaOH. Then, autoclaving of the mixture was done at 115°C and 15 psi for 10 min [21]. Subsequently, the mixture was cooled to room temperature and washed alternately with distilled water and 20 mM sodium acetate buffer (pH 4.3). Final washing was done with sodium acetate buffer (20 mM, pH 4.3). Each washing was followed by centrifugation (5,876*g*, 10 min) till the onset of neutral pH. Subsequently, the residues were dried at 70°C in an oven for 24 h.

### 2.6. FESEM Analysis

25 *μ*L of the untreated and pretreated wild grass (0.05 g·L^−1^) was placed over the glass slide, dried, and coated with gold film using a SC7620“Mini”, Polaron Sputter Coater, Quorum Technologies, Newhaven, England, and analyzed under the field emission scanning electron microscopy (FESEM-Carl Zeiss, SIGMA VP instrument). The images were obtained for the untreated, steam exploded and alkali treated (NaOH) samples of wild grass. 

### 2.7. Simultaneous Saccharification and Fermentation (SSF) Experiments with 1% (w/v) Substrate Concentration at Shake Flask Level

The preliminary SSF trials were performed using recombinant cellulase (GH5) as the hydrolytic enzyme and *S. cerevisiae *as the fermentative organism with all the eight cellulosic substrates to find which substrate gives the best yield of ethanol. 1% (w/v) of each of the eight steam exploded as well as alkali pretreated leafy substrates was autoclaved in 250 mL Erlenmeyer flasks containing 100 mL working volume of sodium acetate buffer (pH 4.3, 20 mM) supplemented with yeast extract (0.1%, w/v) and peptone (0.1%, w/v). To all the eight flasks, 1 mL of crude recombinant cellulase (GH5) (5.4 U·mg^−1^, 0.42 mg·mL^−1^) enzyme isolated by sonication along with *S. cerevisiae* inoculum (3.6 × 10^8^ number of cells·mL^−1^) was added. The flasks were kept at 120 rpm at 30°C in a shaker incubator. The sample was collected at every 6 h interval till 72 h. The dynamic profile of SSF was monitored by measuring the cell OD (*A*_600 nm_), reducing sugar (g·L^−1^), ethanol concentration (g·L^−1^), and specific activity (U·mg^−1^). 

With the aim of hydrolysis of hemicellulose and subsequent utilization of pentose sugars, the next SSF combination involved dual hydrolytic enzyme conglomerate of recombinant cellulase (GH5) and recombinant hemicellulase (GH43) along with a mixture of fermentative organisms.* S. cerevisiae *was the hexose utilizing organism and* C. shehatae *was the pentose utilizing organism. 1% (w/v) of steam exploded wild grass was autoclaved in 250 mL Erlenmeyer flask containing 100 mL working volume of sodium acetate buffer (pH 5.0, 20 mM) supplemented with yeast extract (0.1%, w/v) and peptone (0.1%, w/v). In this case, 0.5 mL of each of isolated recombinant cellulase (GH5) (5.4 U·mg^−1^, 0.42 mg·mL^−1^) and isolated recombinant hemicellulase (GH43) (3.6 U·mg^−1^, 0.28 mg·mL^−1^) was added as the mixture of hydrolytic enzymes. Also, 0.5 mL of each of *S. cerevisiae* (3.4 × 10^9^ cells·mL^−1^) and *C. shehatae* (2.7 × 10^8^ cells·mL^−1^) inoculum was added for fermentation. The fermentation environments along with the estimation of SSF process parameters were similar to single enzyme single culture combination.

### 2.8. SSF Experiment Involving Mixed Enzymes (GH5, GH43) and Mixed Cultures (*S. cerevisiae*, *C. shehatae*) with 5% (w/v) Substrate in Shake Flask and Bioreactor

Subsequently, a higher substrate concentration 5% (w/v) of steam exploded wild grass was used for best SSF combination involving mixed recombinant enzymes GH5 cellulase, GH43 hemicellulase along with mixed cultures (*S. cerevisiae, C. shehatae*). 2.5 mL of each of crude recombinant cellulase (GH5) (5.4 U·mg^−1^, 0.42 mg·mL^−1^) and crude recombinant hemicellulase (GH43) (3.6 U·mg^−1^, 0.28 mg·mL^−1^) for saccharification along with 2.5 mL each of *S. cerevisiae* (3.4 × 10^9^ cells·mL^−1^) and *C. shehatae* (2.7 × 10^8^ cells·mL^−1^) as the fermentative microbes was used for batch SSF at shake flask level. The fermentation medium containing 100 mL of 20 mM sodium acetate buffer and supplemented with 0.1% (w/v) of each of yeast extract and peptone was maintained at initial pH of 5.0. Finally, batch SSF cultivations were performed in a 2 L capacity Bioreactor (Applikon, model Bio Console ADI 1025) with a working volume of 1 L involving mixed enzymes (GH5, GH43) and mixed cultures (*S. cerevisiae, C. shehatae). *5% (w/v) of steam exploded wild grass was used as substrate for SSF studies. 25 mL of each of isolated crude recombinant cellulase (GH5) (5.4 U·mg^−1^, 0.42 mg·mL^−1^) and recombinant hemicellulase (GH43) (3.6 U·mg^−1^, 0.28 mg·mL^−1^) for saccharification along with 25 mL each of *S. cerevisiae* (3.4 × 10^9^ cells·mL^−1^) and *C. shehatae* (2.7 × 10^8^ cells·mL^−1^) for bioethanol production was employed for fermentation experiments. The temperature at 30°C, pH at 5.0, and agitation of 120 rpm were maintained. The aeration rate was controlled at 1 vvm by a mass flow controller to maintain dissolved oxygen (DO) level of minimum 40% for the efficient growth of fermentative microbes. Growth was monitored at 600 nm using spectrophotometer (Varian Cary50, Australia). The online process parameters like temperature (°C), pH, and stirring rate (rpm) were monitored and recorded for every 1 min. The various parameters such as cell OD (*A*_600 nm_), reducing sugar (g·L^−1^), ethanol concentration (g·L^−1^), and specific activity (U·mg^−1^) were examined at fixed intervals of 6 h. The pH was upheld at a set point of 4.3 by addition of 1 N HCl and 1 N NaOH. Thus, pH excursions of the organism below the set point were not permitted owing to its sensitivity for such changes. After the completion of SSF process, filtration of the fermentation broth was done and the filtrate collected was subjected to further recovery by vacuum evaporation.

### 2.9. Recovery of Partially Purified Ethanol

The filtered fermentation broth containing bioethanol was concentrated under vacuum in a rotary evaporator (Buchi Rotavapor R-200, Switzerland). The process was carried out in a 2 L round bottom evaporation flask encompassing 1 L working volume of fermentation broth. The heating was done in a water bath (Buchi Heating Bath B-490) for 3 h at 78.5°C. Finally, the distillate containing the partially purified ethanol was collected and estimated by dichromate method [22] as described later. 

The purification process efficiency of ethanol obtained by rotary evaporator was calculated using the following equation:

(1) purification  process  efficiency  (%)=volume  of  partially  purified  ethanol  in  distillate(mL/L)crude  ethanol  in  fermentation  broth(mL/L) ×100.

### 2.10. Analytical Methods

#### 2.10.1. Cellulose, Hemicellulose, and Lignin Estimation

The structural carbohydrates like cellulose, hemicellulose, and lignin were estimated by standardized methods of NREL, USA [23]. 0.3 g of dry substrate (lignocellulosic leafy biomass) was mixed with 3 mL of 27 N H_2_SO_4_ and incubated at 30°C for 1 h. Then 84 mL of distilled water was added to lower down H_2_SO_4_ concentration to 1.5 N. The sample was autoclaved at 121°C for 1 h. The substrate was cooled to room temperature and the treated biomass was filtered using a vacuum filtration unit. The residue was weighed which was lignin (Acid Insoluble Lignin). The filtrate was collected and pH was neutralized by addition of 1 M CaCO_3_. Finally, the filtrate was assayed for reducing sugar which is glucose from where cellulose is calculated. (1 g cellulose = 1.1 g of glucose). The remaining content is hemicellulose.

#### 2.10.2. High Pressure Anion Exchange Chromatography (HPAEC) Analysis of Polysaccharides Hydrolyzed by GH5 Cellulase and GH43 Hemicellulase

High pressure anion exchange chromatography (HPAEC) was executed to detect monosaccharides released by enzymatic degradation of complex polysaccharides from wild grass (*Achnatherum hymenoides*) during SSF using CARBOPACK^TM^ PA-20 column (Dionex) as described by Van Gool et al. [24] with modification in flow rate. The instrument (ICS-3000, Dionex) was kept at 30°C with a loop size of 25.0 *μ*L and flow rate of 0.5 mL·min^−1^ throughout the analysis. The elution of reducing sugars was performed with 100.0 mM NaOH and analyzed by pulsed amperometric detector (PAD) in tandem with Dionex (ICS-3000). The HPAEC profiles of the hydrolyzed product, glucose by GH5 cellulase, and arabinose by GH43 hemicellulase were studied at 0, 18, 36, 54, and 72 h, respectively. Arabinose, glucose, and xylose were used as standard (1.2 mg·mL^−1^ final concentration of each sugar in the standard mixture). The crude sample (200 *μ*L) was diluted with 400 *μ*L of ultrapure water and centrifuged at 15,493*g* for 15 min. The supernatant (500 *μ*L) was filtered through 0.2 *μ*m membrane and subsequently injected into HPAEC-PAD. 

#### 2.10.3. Enzyme Assay and Protein Content

The GH5 cellulase assay was carried out by incubating the 10 *μ*L enzyme with 1% (w/v) final concentration of CMC in 20 mM sodium acetate buffer (pH 4.3) in a 100 *μ*L reaction mixture at 50°C for 10 min. The mixture was analysed for the release of reducing sugar [18, 19]. The enzyme activity was calculated by measuring the released reducing sugar. The hemicellulase GH43 activity was assayed by incubating the 10 *μ*L enzyme with 1% (w/v) final concentration of rye arabinoxylan in 100 mM sodium acetate buffer (pH 5.4) in a 100 *μ*L reaction mixture at 50°C for 10 min. The absorbance was measured at 500 nm against a blank with D-glucose as standard using a UV-visible spectrophotometer (Perkin Elmer, Model lambda-45). One unit (U) of cellulase activity is defined as the amount of enzyme liberating 1 *μ*mole of reducing sugar (glucose) per min under the previous assay conditions. One unit (U) of hemicellulase activity is defined as the amount of enzyme liberating 1 *μ*mole of reducing sugar (arabinose) per min under the previous assay conditions. The protein concentration was measured by using 10 *μ*L of enzyme along with 90 *μ*L of distilled water in 100 *μ*L of total reaction volume with the addition of 1 mL of Bradford reagent [25]. The reaction mixture was maintained at 25°C for 20 min and OD at 595 nm was determined using a UV-visible spectrophotometer (Perkin Elmer, Model lambda-45). BSA was used as standard.

#### 2.10.4. Ethanol Estimation by Gas Chromatography and Dichromate Assay

Ethanol was analyzed by gas chromatography equipped with flame ionization detector (GC-FID, Varian 450) and column packed with Porapak (Hayesep) Q (3.0 m × 2.0 mm i.d., 80–100 mesh, manufactured by Varian) [26]. Nitrogen at a constant flow rate of 55 cm^3^·min^−1^ was used as the carrier gas, and the oven temperature was kept isothermally at 150°C for 20 minutes. The injector and detector temperatures were kept at 170°C, and the injection volume of 1 *μ*L was used for analysis.

 Ethanol content was also assessed by its conversion to acid by dichromatic reaction following the method of Seo et al. [22]. 1 mL of the cell free supernatant was mixed with 2 mL of K_2_Cr_2_O_7_ (0.115 M) and 9 mL of distilled water. The reaction mixture (12 mL) was maintained in a boiling water bath for 10 min. Finally, the sample was cooled and the absorbance was measured at 600 nm against a blank with dichromate as standard using a UV-visible spectrophotometer (Perkin Elmer, Model lambda-45).

## 3. Results and Discussion

The technoeconomic feasibility of lignocellulosic ethanol fermentation by SSF process depends on efficient consumption of both monomeric sugars derived from complex cellulose and hemicellulose moieties of various agricultural residues. Release of hexoses and pentoses from varied range of leafy substrates with structural polysaccharide contents require a combination of saccharifying enzymes. Further, an efficient pretreatment method for the lignin content removal with accessibility to hydrolytic enzymes also becomes essential to enrich the availability of utilizable forms of reducing sugars from substrates. In the current research, efficacy of implementing recombinant cellulase (GH5) and hemicellulase (GH43) from *C. thermocellum* expressed in different *E. coli* BL21 strains was analyzed for hexoses and pentoses production, respectively, which was further utilized for ethanol production from several leafy biomasses using a mixed culture of *S. cerevisiae *and *C. shehatae.*

### 3.1. Production of Recombinant Hydrolytic Enzymes from *E. coli* by Batch and Repetitive Batch Fermentation

**Figure 1:**
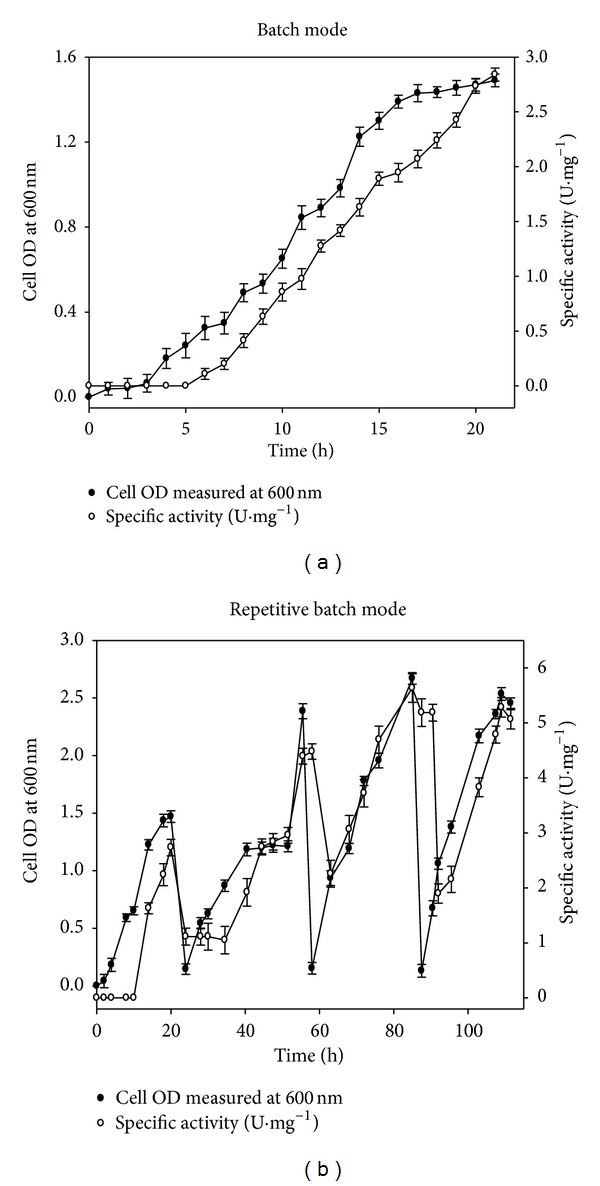
Dynamic profile of cell growth and recombinant cellulase (GH5) activity. (a) Batch mode and (b) repetitive batch mode. (●) cell OD measured at 600 nm and (○) specific activity (U·mg^−1^) with time, respectively.

The consequence of batch and repetitive batch mode operations on the synthesis of recombinant cellulase and hemicellulase was studied at shake flask level. The dynamic profiles of cell biomass (*A*_600 nm_) and specific activity (U·mg^−1^) of recombinant cellulase (GH5) in the batch and repetitive batch mode, respectively, are shown in Figures 1(a) and 1(b). A constant volume repetitive batch operation was designed which used the IPTG induced *E. coli* cells as the inoculum for the subsequent batch operation. In batch mode, the maximum enzyme activity and the cell OD (*A*_600 nm_) obtained were 2.8 U·mg^−1^ and 1.4, respectively (Figure 1(a)). In the second cycle of repetitive batch fermentation, a 1.5-fold increase in both specific enzyme activity and cell OD (*A*_600 nm_) was observed while being compared with the batch mode fermentation (Figure 1(b)). Finally, a 2-fold increment in both biomass productivity (*A*_600 nm_ = 2.8) and enzyme activity (5.6 U·mg^−1^) was observed in third cycle of repetitive batch mode when being compared with batch mode production of recombinant cellulase (GH5) (Table 1, Figure 1(b)), respectively. A similar observation was recorded for the synthesis of recombinant hemicellulase by cultivating *E. coli* BL21 cells harbouring GH43 gene in both modes. In batch mode, the maximum enzyme activity of 2.2 U·mg^−1^ and the highest cell OD (*A*_600 nm_) of 1.6 (Table 1) were obtained whereas in repetitive 3rd batch the maximum specific activity and the cell density obtained were 3.8 U·mg^−1^ and 2.9, respectively (Table 1). Overall enzyme synthesis and their activities depend upon the IPTG induction of *lac* constitutive system and the cell biomass productivity. The cell biomass increased to a higher extent owing to the consecutive use of induced cells in the following repetitive batches increasing the productivity of enzyme during repetitive batch fermentation. The biomass production of cells is inhibited by acetate toxicity which retards the recombinant proteins' expression, and consequently, the cell density owing to the variations in pH, oxygen availability, and limited substrate availability [27]. Repetitive batch operation is a robust alternative, where the cells are maintained in active exponential growth phase by the use of induced inoculum in subsequent batches.

### 3.2. Composition Analysis of Agricultural Substrates

The composition of raw substrates involved in the present study is shown in Table 2. The maximum cellulose content was observed in wild grass (51%, w/w) followed by jamun (40%, w/w), bamboo (37%, w/w) and the lowest in neem (20%, w/w) (Table 2). The maximum hemicellulose content was found in mango (54%, w/w) followed by neem (52%, w/w), poplar (49%, w/w), and eucalyptus (47%, w/w) (Table 2). The stovers of Bermudagrass, reed, and rapeseed were reported to contain cellulose content of 47.8%, 39.5%, and 27.6% and lignin content of 19.4%, 24.0%, and 18.3%, respectively (all values are in w/w) [28]. Cellulose content of rice husk reported was 30 (%, w/w) which is lower than the cellulose contents of various substrates used in the present study [29]. Among eight substrates, wild grass containing the highest cellulose content 51% (w/w) with 30% (w/w) hemicellulose was selected as the most sustainable substrate for bioethanol production.

### 3.3. Substrate Pretreatment and SSF in Shake Flask

**Figure 2:**
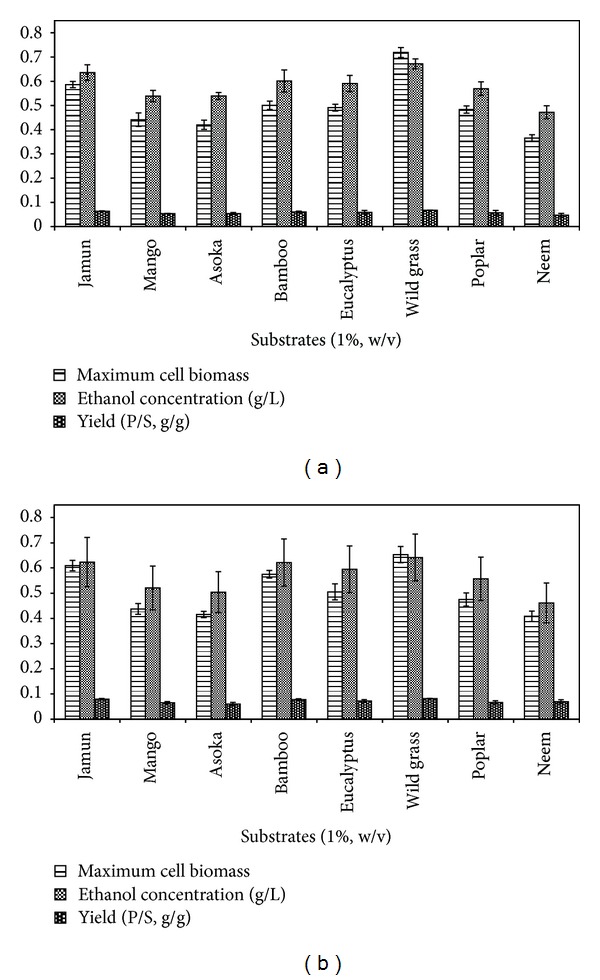
Effect of pretreatments. (a) Steam explosion and (b) alkali (NaOH) pretreatment on eight substrates subjected to simultaneous saccharification and fermentation trials.

**Figure 3:**
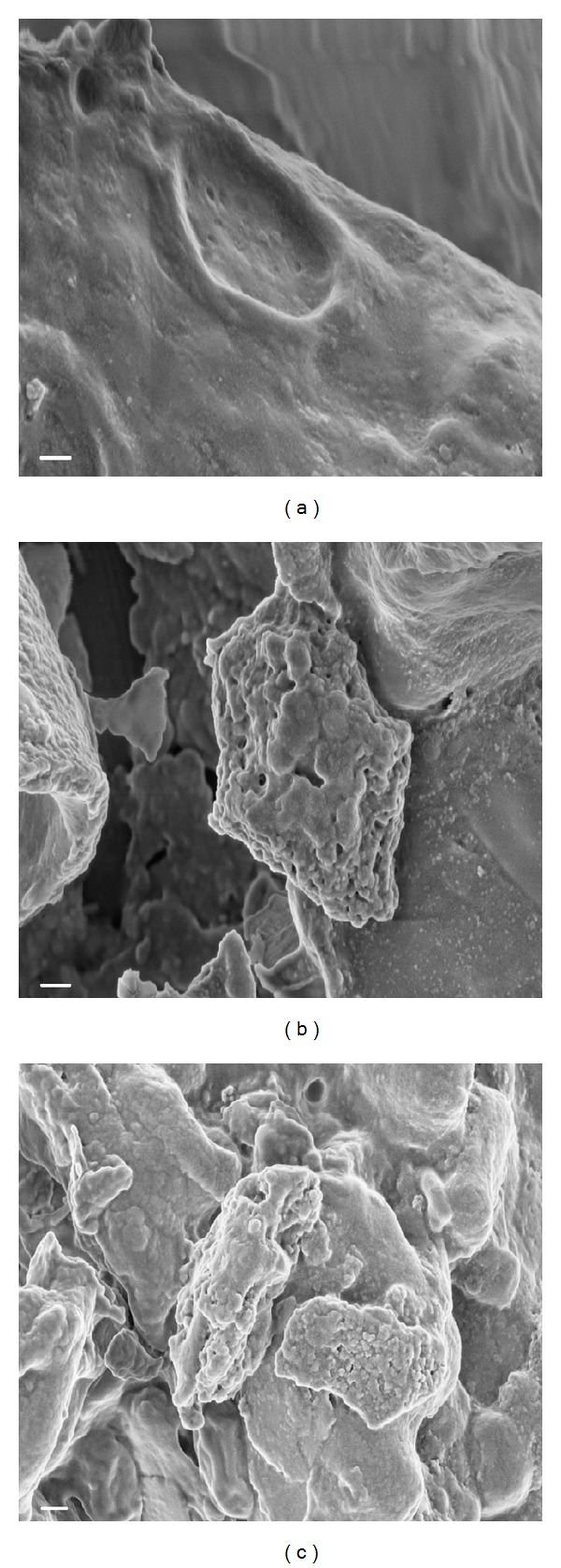
Representative FESEM images. (a) Untreated wild grass, (b) steam exploded wild grass, (c) alkali (NaOH) treated wild grass. All images are shown at same magnification-scale bar: 200 nm. Topological changes associated with pretreatment, namely, steam explosion and alkali based treatment are clearly detectable in (b) and (c).

The lignocellulosic substrates were subjected to various pretreatment methods for increasing the efficiency of saccharification. The current study assessed the steam explosion and alkali (NaOH) pretreatment effects on lignocellulosic substrates in terms of total reducing sugar and ethanol titre (Figures 2(a) and 2(b)). All the eight pretreated substrates were subjected to simultaneous hydrolysis and fermentation using recombinant cellulase (GH5) as the saccharifying enzyme and *S. cerevisiae *as the bioethanol producer to determine the most appropriate pretreatment strategy and the best substrate for ethanol fermentation. A maximum ethanol concentration was achieved for wild grass (0.67 g·L^−1^) from maximum reducing sugar concentration (1.26 g·L^−1^) with a yield coefficient of 0.067 (g of ethanol·g of substrate^−1^) followed by jamun (0.63 g·L^−1^ ethanol, reducing sugar 1.02 g·L^−1^, yield coefficient 0.063 g·g^−1^) employing steam explosion pretreatment (Figure 2(a)). In another experiment involving alkali pretreated substrates, 0.65 g·L^−1^ of ethanol was obtained in wild grass from a reducing sugar concentration of 1.20 g·L^−1^ with an ethanol yield of 0.065 g·g^−1^, while jamun produced 0.60 g·L^−1^ of ethanol from 0.99 g·L^−1^ reducing sugar, with the yield coefficient being 0.060 g·g^−1^ (Figure 2(b)). The two pretreatment methods were more or less similar in their efficiencies. FESEM images were obtained for the untreated, steam exploded and alkali treated (NaOH) wild grass (Figures 3(a), 3(b), and 3(c)). The surface of the untreated substrate was found to be nonporous and structurally even (Figure 3(a)). The structural destabilization and the surface porosity of wild grass were more pronounced after steam explosion (Figure 3(b)) than after alkali (NaOH) pretreatment (Figure 3(c)). The structural stability changes and increased porosity rendered the cellulosic and hemicellulosic contents of the substrate more accessible for hydrolysis hence proving an efficient pretreatment process mandatory. The conformation study of the sugars in the substrates before and after the treatment strategies suggested that steam explosion is the best pretreatment method.

### 3.4. SSF Involving Recombinant Cellulase (GH5) as Hydrolytic Enzyme and *S. cerevisiae* as Fermentative Microbe with 1% (w/v) Steam Exploded Wild Grass at Shake Flask Level

**Figure 4:**
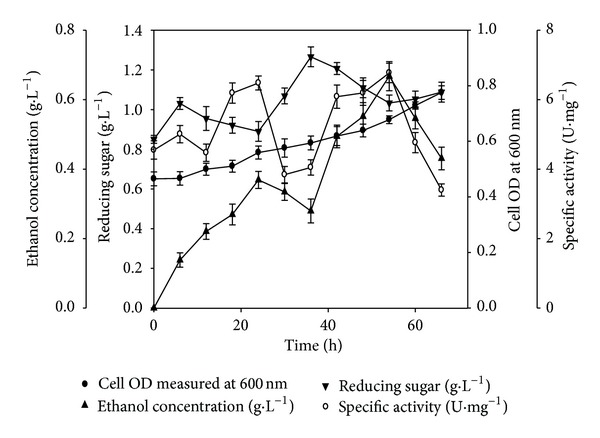
SSF profile of 1% (w/v) wild grass using GH5 cellulase, *S. cerevisiae *in shake flask. (●) cell OD measured at 600 nm, (▲) ethanol concentration (g·L^−1^), (*▼*) reducing sugar (g·L^−1^), and (○) specific activity (U·mg^−1^) with time (h). The cultivation conditions were 100 mL working volume in 250 mL Erlenmeyer flask, initial pH 4.3, temperature 30°C, and shaking 120 rpm.

In the initial experiments, the untreated wild grass gave an ethanol titre of 0.70 g·L^−1^ as compared to steam exploded wild grass that gave higher titre value of 0.82 g·L^−1^ of ethanol. Among the eight substrates employed in fermentation trials, wild grass (*Achnatherum hymenoides)* offered the best results both in terms of ethanol titre and yield. The findings suggested that steam exploded wild grass was the potential substrate for ethanol production. SSF experiment was performed involving 1% (w/v) steam exploded wild grass as substrate and recombinant cellulase (GH5) as saccharifying enzyme along with *S. cerevisiae* for bioethanol production. The dynamic profile of SSF exhibited three distinct phases in terms of growth of fermentative microbes, specific activity of enzyme, release of utilizable sugar, and rate of ethanol formation (Figure 4). In the first phase, there was a drop in reducing sugar after a short initial accumulation phase. A decrease in sugar concentration was found to be concomitant with simultaneous increase in growth and ethanol concentration. In the second phase of fermentation, the organisms were still in their exponential phase reaching a maximum OD of 0.8 at 66 h. Interestingly, a decrease in ethanol concentration was observed with the substantial accumulation of reducing sugar (1.26 g·L^−1^) in the broth (Table 3, Figure 4). The third and final phase of fermentation was marked with a steep rise in ethanol concentration attaining a maximum titre of 0.67 g·L^−1^ and yield of 0.067 (g of ethanol·g of substrate^−1^) (Table 3, Figure 4). This phase displayed a continuous sugar withdrawal from the broth. There was a drop in cell biomass concentration after 66 h indicating achievement of senescence. The dynamic profile of reducing sugar showed a sinusoidal behaviour attributing to a delicate balance between the rate of saccharification for reducing sugar release and the extent of its utilization for growth and ethanol formation. It was found that the reducing sugar concentration increment was associated with decline in specific enzyme activity and vice versa. 

### 3.5. SSF Involving Mixed Enzymes (GH5, GH43) and Mixed Cultures (*S. cerevisiae*, *C. shehatae*) Using 1% (w/v) Steam Exploded Wild Grass in Shake Flask

**Figure 5:**
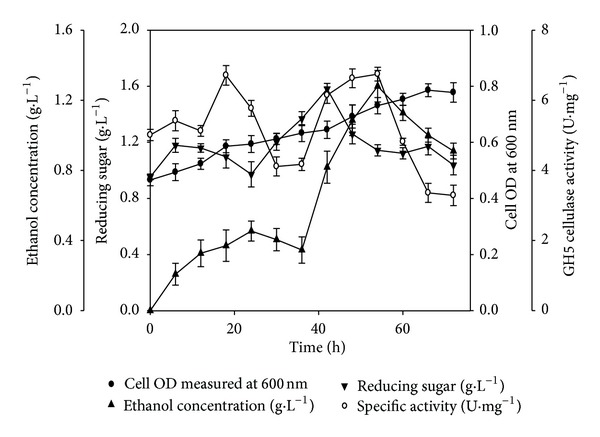
SSF profile of 1% (w/v) wild grass using mixed enzyme mixed culture in shake flask. (●) cell OD measured at 600 nm, (▲) ethanol concentration (g·L^−1^), (*▼*) reducing sugar (g·L^−1^), and (○) specific activity (U·mg^−1^) with time (h). The cultivation conditions were 100 mL working volume in 250 mL Erlenmeyer flask, initial pH 5.0, temperature 30°C, and shaking 120 rpm. The mixed hydrolytic enzymes were GH5 cellulase with GH43 hemicellulase, and mixed fermentative microbes were* S. cerevisiae *with* C. shehatae. *The dynamic profiles of recombinant cellulase (GH5) and hemicellulase (GH43) (data not shown) were similar.

The structural composition analysis of different substrates revealed that the collective cellulose and hemicellulose fractions contributed towards approximately 80% (w/w) of the total dry biomass. With the objective of utilizing both polysaccharide fractions and to validate the hypothesis that bioethanol can also be derived from pentose sugars [11, 30], subsequent shake flask level SSF experiments were investigated involving a combination of mixed cultures and mixed enzymes. The mixed enzyme system consisted of a mixed consortium of recombinant cellulase (GH5) and hemicellulase (GH43) for hydrolysis of cellulose and hemicellulose fractions, respectively. The mixed culture system involved *S. cerevisiae* and *C. shehatae* with the aim of utilizing hexose and pentose sugars, respectively. The microorganisms did not exhibit any lag phase in their growth as evident from Figure 5. The growth was steady till the end of the experiment until 66 h, but after that, a slight decrease was observed in growth. Figure 5 represented biphasic ethanol fermentation kinetics. The initial phase of ethanol synthesis occurred till 24 h from the onset of SSF. The final phase of ethanol fermentation started from 36 h producing a maximum ethanol titre of 1.28 g·L^−1^ (Table 3, Figure 5) with a yield of 0.128 (g of ethanol·g of substrate^−1^) at 54 h only to decrease thereupon. The initial phase of the SSF depicted an accumulation of available sugars up to 6 h. There was a fall in reducing sugar concentration from 6 h to 24 h; after that, the sugar concentration increased till 48 h, where it reached a maximum value of 1.60 g·L^−1^ and then declined (Figure 5). Interestingly, the sinusoidal behaviours of enzyme activities of GH5 cellulase and GH43 hemicellulase and reducing sugars shared an inverse relationship throughout the SSF experiment. As wild grass contains more cellulose, the dynamic profile of only recombinant cellulase (GH5) has been shown (Figure 5). The mixed enzymatic consortium of GH5 cellulase and GH43 hemicellulase along with mixed cultures (*S. cerevisiae*, *C. shehatae*) resulted in twofold upturn both in ethanol titre and yield (Table 3, Figure 5) as compared to single enzyme (GH5 cellulase) and single culture (*S. cerevisiae*) SSF experiments (Table 3, Figure 4). The use of GH43 hemicellulase released more amounts of utilizable pentose sugars along with hexoses by GH5 cellulase giving an improved ethanol titre as compared to recombinant cellulase (GH5) releasing only hexose sugars.

### 3.6. SSF Involving Mixed Recombinant Enzymes (GH5, GH43) and Mixed Cultures (*S. cerevisiae*, *C. shehatae*) Using 5% (w/v) Steam Exploded Wild Grass in Shake Flask and Bioreactor

Increasing the substrate concentration along with enzyme loadings and inoculum is reported to enhance ethanol titre and yield [31]. The batch SSF was executed in shake flask using a 5% (w/v) substrate concentration along with mixed enzymes and mixed cultures. The mixed consortium of enzymes and fermentative microbes yielded ethanol concentration of 11.2 g·L^−1^ with a maximum released reducing sugar concentration of 13.0 g·L^−1^ with ethanol yield of 0.224 (g of ethanol·g of substrate^−1^) (Table 3). Therefore, an increase in substrate concentration to 5% (w/v) wild grass yielded a 8.7-fold increase in ethanol titre and 1.7-fold increase in ethanol yield, as compared to 1% (w/v) substrate concentration in shake flask (Table 3). 

**Figure 6:**
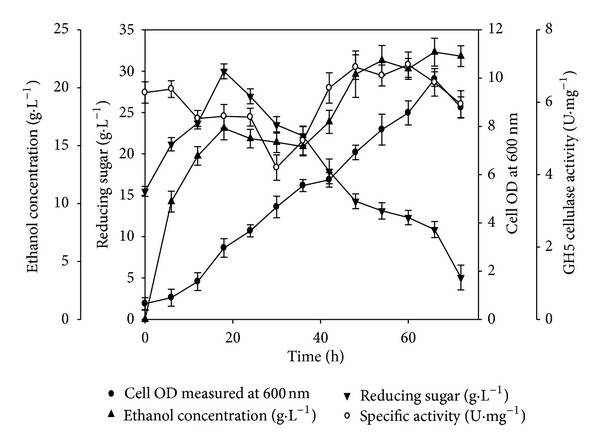
SSF profile of 5% (w/v) wild grass using mixed enzyme mixed culture in a bioreactor. (●) cell OD measured at 600 nm, (▲) ethanol concentration (g·L^−1^), (*▼*) reducing sugar (g·L^−1^), and (○) specific activity (U·mg^−1^) with time (h). The cultivation conditions were 1 L working volume in 2 L lab scale bioreactor with aeration rate 1 vvm, pH 5.0, temperature 30°C, and agitation 120 rpm. The mixed hydrolytic enzymes were GH5 cellulase with GH43 hemicellulase and mixed fermentative microbes were* S. cerevisiae *with* C. shehatae. *The dynamic profiles of recombinant cellulase (GH5) and hemicellulase (GH43) (data not shown) were similar.

**Figure 7:**
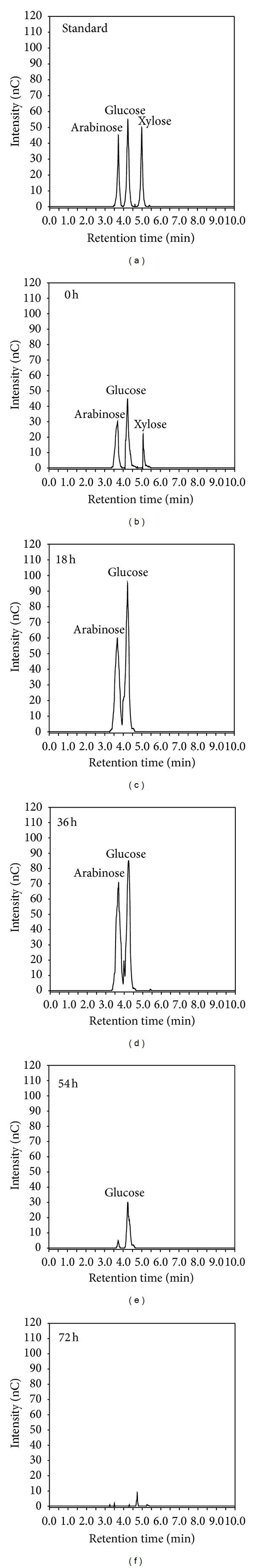
Typical HPAEC pattern of monosaccharides obtained from bioreactor SSF of 5% (w/v) wild grass. The chromatograms of sugar hydrolysate, namely, arabinose, glucose, and xylose were obtained at different time intervals by HPAEC-PAD (high pressure anion exchange chromatography pulsed amperometric detector), (a) Standard, (b) 0 h, (c) 18 h, (d) 36 h, (e) 54 h, and (f) 72 h.

The performance of trials in an automated bioreactor makes the stringent monitoring of important process parameters possible. The parameters, pH, and aeration significantly affect the fermentation dynamics and in turn the final ethanol titre [32]. The SSF using 5% (w/v) steam exploded wild grass was scaled up in the lab scale 2 L bioreactor. The fermentative organisms followed an exponential growth profile, remaining in the lag phase for initial 6 h (Figure 6). There was an increase in biomass concentration as the organisms entered the log phase until the 66 h, then reached its maximum cell OD (*A*_600 nm_) of 10, and finally followed a decline in the growth. Ethanol formation was recorded in two distinct phases. The first phase of ethanol production recorded a titre of 16 g·L^−1^ at 18 h of fermentation followed by a slight decrease in the rate of ethanol synthesis till 36 h. The second and final phase of fermentation witnessed a maximum ethanol titre of 23.1 g·L^−1^ with an ethanol yield of 0.461 (g of ethanol·g of substrate^−1^) at 54 h and then showed decline in ethanol titre till the end of the SSF process (Table 3, Figure 6). The reducing sugar concentration escalated during the initial 18 h reaching a maximum value of 30.0 g·L^−1^ (Table 3, Figure 6). The dynamic profile of only recombinant cellulase (GH5) has been shown in Figure 6 as wild grass contains more cellulose. The uptake of sugar by the fermentative microbes for their growth, maintenance, and production of ethanol after 36 h accounted for the drop in sugar concentration for the rest of the period of fermentation process. The dynamic profile of SSF exhibited an inverse relationship between rates of sugar utilization and ethanol formation. The degradation products released from cellulosic and hemicellulosic content of wild grass by saccharification in a bioreactor were detected by HPAEC-PAD (Figures 7(a), 7(b), 7(c), 7(d), 7(e), and 7(f)). The retention time for different monosaccharide sugars arabinose, glucose and xylose used as standard was 3.71, 4.23, and 4.98 min, respectively (Figure 7(a)). The HPAEC profile of the monosaccharides at 0 h witnessed little amount of xylose that might have released along with arabinose and glucose during pretreatment (Figure 7(b)). Surprisingly, no xylose was detected in the subsequent stages of SSF due to its uptake by *C. shehatae *(Figures 7(c), 7(d), 7(e), and 7(f)). The 18 h HPAEC profile detected substantial amount of glucose and arabinose released by mixed enzyme consortium (GH5 cellulase, GH43 hemicellulase) (Figure 7(c)). The HPAEC profile at 36 h also exhibited considerable amount of monosaccharide sugars (Figure 7(d)). During the 54 h, the HPAEC profile exhibited little amount of glucose with trace quantity of arabinose due to their utilization by *S. cerevisiae* and *C. shehatae* for ethanol formation (Figure 7(e)). The HPAEC pattern at 72 h showed negligible amount of monosaccharide sugars due to their consumption by fermentative yeasts for growth, maintenance, and ethanol formation (Figure 7(f)).

 The controlled conditions of pH 5.0 and aeration rate significantly affected the growth and ethanol titre. 1 vvm aeration rate was kept to maintain a threshold dissolved oxygen (DO) level of minimum 40% for the efficient growth of fermentative organisms and in turn a good product yield. A 2-fold increase in ethanol titre (23 g·L^−1^) (Table 3) was obtained in lab scale bioreactor on scaling up the shake flask SSF (11.2 g·L^−1^) (Table 3) with mixed enzyme mixed culture system using 5% (w/v) wild grass. Similarly, 16.5 g·L^−1^ ethanol with yield of 0.33 (g of ethanol·g of substrate^−1^) was reported from 5% (w/w) corncob [31]. Addition of *C. shehatae* along with *S. cerevisiae* increased the overall ethanol yield as the pentose sugars released from lignocellulosic hydrolysis were metabolized by *C. shehatae*. 

The dynamic profiles of multiple offline measurements from various batch runs demonstrated a complex interplay between the saccharification rates of lignocellulosic substrates, sugar utilization, and in turn ethanol formation. The reducing sugar profile represents a sinusoidal behavior indicating a gentle balance between the hydrolysis rate and the utilization rate of reducing sugars for growth and ethanol fermentation. The accumulation of sugar in the broth resulted in enzyme inhibition which in turn lessened the rate of hydrolysis. Similarly, a 75% repressive effect on the activity of cellulase was reported by a glucose concentration of 20 g·L^−1^ [33]. A diminution in reducing sugar content following an accumulation is a collective effect of reduction in rate of hydrolysis plus growth energy metabolism and in turn ethanol formation. A depletion of sugar was witnessed without any additional increase in ethanol titre during the late log phase indicating that the sugars were utilized only for upkeep and survival of the microbes. 

### 3.7. Recovery of Partially Purified Ethanol and Purification Process Efficiency Determination

The ethanol from fermentation broth was recovered using a rotary vacuum evaporator. The crude ethanol obtained in SSF studies employing mixed recombinant enzymes (GH5 cellulase, GH43 hemicellulase) and mixed cultures (*S. cerevisiae, C. shehatae*) using 5% (w/v) steam exploded wild grass at bioreactor level was 29.15 mL·L^−1^, that is, 23 g·L^−1^. 1 L fermentation broth with crude ethanol on vacuum evaporation yielded 6.75 mL of distillate containing 6.18 mL, that is, 91.6% (v/v) of partially purified ethanol. Finally, the efficiency of purification was estimated to be 21.23% [12]. The remaining ethanol in the broth obtained with the water condensates can be recovered through repeated distillation. In the large-scale operations, multiple distillation steps are included to obtain 100% recovery from the water condensates [12]. The minimum evaporation loss in the condensate collector with maximum recovery can be achieved by a rotary vacuum evaporator equipped with multiple condenser units. The values of ethanol titre attained in our studies are analogous with other values reported in the literature. A coculture of *Clostridium thermosaccharolyticum *HG8 and *Thermoanaerobacter ethanolicus *ATCC 31937 was reported to yield an ethanol concentration of 2.2 g·L^−1^ from 1% (w/v) of banana waste [34]. An ethanol concentration (2.1 g·L^−1^) was reported using commercial cellulolytic enzyme and 1% (w/v) sunflower stalks [35]. Zhang et al. reported an ethanol titre of 62.7 g·L^−1^ using 19% (w/w) dry corncorb and commercial cellulolytic enzymes in a bioreactor [31]. Wheat straw (5%, w/v) yielding an ethanol titre of 5 g·L^−1^ was reported using crude unprocessed *Trichoderma reesei* cellulase [36]. An ethanol concentration (3.36 g·L^−1^) was reported from 50 g·L^−1^ pretreated sugarcane bagasse under optimized process conditions in aerobic batch fermentation in a lab scale reactor [37]. An ethanol titre of 1.4 g·L^−1^ employing recombinant *Clostridium thermocellum *cellulase and *S. cerevisiae *from 1% (w/v) Jamun (*Syzygium cumini*) leafy substrates was reported [6]. This exhibits that usage of mixed culture system involving economically feasible mixed recombinant enzymes, GH5 cellulase and GH43 hemicellulase in the present SSF studies offers a comprehensive choice of bioethanol production.

## 4. Conclusions

Bioethanol production employing recombinant *Clostridium thermocellum* enzymes was investigated. Enhanced production of recombinant cellulase and hemicellulase was obtained with repetitive batch mode. Wild grass proved to be the best substrate among other leafy biomasses. Steam exploded wild grass gave higher ethanol titre as compared to alkali pretreatment. The mixed enzyme and mixed culture system using 1% (w/v) pretreated wild grass yielded 2-fold higher ethanol than single enzyme single culture system. A 5% (w/v) rise in substrate concentration along with enzyme loadings and inoculum volume from shake flask to a lab scale bioreactor yielded a 2-fold increase both in ethanol titre and yield. In brief, SSF processes based on recombinant enzymes have the potential to maximize the volumetric efficiency while curtailing the production expenses in the bioethanol industry. 

## Figures and Tables

**Table 1:** Overall data values for biomass productivity and specific enzyme activity.

Enzyme type	Mode	Maximum biomass* (cell OD)	Specific activity* (U·mg^−1^)
GH5 cellulase	Batch	1.4 ± 0.04	2.8 ± 0.07
	Repetitive batch	2.8 ± 0.05	5.6 ± 0.08
GH43 hemicellulase	Batch	1.6 ± 0.03	2.2 ± 0.04
	Repetitive batch	2.9 ± 0.06	3.8 ± 0.06

*Values are mean ± SE (*n* = 3).

**Table 2:** Cellulose, hemicellulose, and lignin content (%) of various lignocellulosic leafy biomasses.

Substrates (leafy biomass)	Cellulose* (%)	Hemicellulose* (%)	Lignin* (%)
Wild grass (*Achnatherum hymenoides*)	51.23 ± 0.43	30.06 ± 0.55	18.70 ± 0.56
Jamun (*Syzygium cumini*)	40.36 ± 0.45	32.22 ± 0.52	27.40 ± 0.49
Bamboo (*Bambusa dendrocalamus*)	37.30 ± 0.50	35.04 ± 0.47	27.65 ± 0.42
Eucalyptus (*Eucalyptus marginata*)	35.68 ± 0.49	47.44 ± 0.50	16.87 ± 0.45
Poplar (*Populus nigra*)	29.40 ± 0.40	48.84 ± 0.38	21.75 ± 0.46
Mango (*Mangifera indica*)	27.16 ± 0.38	53.98 ± 0.43	18.85 ± 0.50
Asoka (*Saraca indica*)	26.62 ± 0.32	30.06 ± 0.50	21.81 ± 0.50
Neem (*Azadirachta indica*)	20.64 ± 0.44	50.84 ± 0.48	18.52 ± 0.40

*Values are mean ± SE (*n* = 3).

**Table 3:** Different SSF combinations of recombinant hydrolytic enzymes and fermentative microbes with steam exploded wild grass.

SSF combination	Substrate concentration (%, w/v) and mode of SSF	Reducing sugar* (g·L^−1^)	Ethanol yield (g of ethanol·g of substrate^−1^)	Ethanol titre* (g·L^−1^)
GH5 + *S. cerevisiae *	1% shake flask	1.26 ± 0.06	0.067	0.67 ± 0.02
GH5 + GH43 + *S. cerevisiae* + *C. shehatae* (shake flask)	1% shake flask	1.60 ± 0.05	0.128	1.28 ± 0.03
GH5 + GH43 + *S. cerevisiae* + *C. shehatae* (shake flask)	5% shake flask	13.0 ± 0.04	0.224	11.20 ± 0.06
GH5 + GH43 + *S. cerevisiae* + *C. shehatae* (bioreactor)	5% bioreactor	30.0 ± 0.04	0.461	23.07 ± 0.08

*The values correspond to the maximum reducing sugar and maximum ethanol at a particular time; values are mean ± SE (*n* = 3).

## Abbreviations

SSF: Simultaneous saccharification and fermentation
*A*. *hymenoides*: *Achnatherum hymenoides*
*T. reesei*: *Trichoderma reesei*
*C. thermocellum*: *Clostridium thermocellum*
GH5: Glycoside hydrolase family 5
GH43: Glycoside hydrolase family 43
*E. coli*: *Escherichia coli*
*S. cerevisiae*: *Saccharomyces cerevisiae*
*C. shehatae*: *Candida shehatae*
g·L^−1^: Gram per litre
HPAEC-PAD: High pressure anion exchange chromatography-pulsed amperometric detector
*A*_600 nm_: Optical density at 600 nm
GC: Gas chromatography
Yield of ethanol (g·g^−1^):^−1^: Yield of ethanol (gram of ethanol·gram of substrate).
